# Synthesis, Adsorption Isotherm and Kinetic Study of Alkaline- Treated Zeolite/Chitosan/Fe^3+^ Composites for Nitrate Removal from Aqueous Solution—Anion and Dye Effects

**DOI:** 10.3390/gels8120782

**Published:** 2022-11-29

**Authors:** Endar Hidayat, Tomoyuki Yoshino, Seiichiro Yonemura, Yoshiharu Mitoma, Hiroyuki Harada

**Affiliations:** 1 Graduate School of Comprehensive and Scientific Research, Prefectural University of Hiroshima, Shobara 727-0023, Japan; 2 Faculty of Bioresources Science, Department of Life and Environmental Science, Prefectural University of Hiroshima, Shobara 727-0023, Japan

**Keywords:** adsorption, chitosan, Fe^3+^, nitrate removal, zeolite

## Abstract

In the present study, alkaline-treated zeolite/chitosan/Fe^3+^ (ZLCH-Fe) composites were prepared and analyzed using scanning electron microscopy (SEM), Fourier-transform infrared spectroscopy (FTIR) and pH of zero point of charge (pH_zpc_) to remove nitrates from water. The process was carried out using an adsorption method with a varied initial pH, adsorbent dosage, initial nitrate concentration and contact time. The pH_zpc_ demonstrated that the ZLCH-Fe surface had a positive charge between 2 and 10, making it easier to capture the negative charge of nitrate. However, the optimal pH value is 7. After 270 min, the maximum adsorption capacity and percent removal reached 498 mg/g and 99.64%, respectively. Freundlich and pseudo-second-order were fitted to the adsorption isotherm and kinetic models, respectively. An evaluation was conducted on the effects of anions—SO_4_^2−^ and PO_4_^3−^—and dyes—methylene blue (MB) and acid red 88 (AR88)—upon nitrate removal. The results indicated that the effect of the anion could be inhibited, in contrast to dye effects. However, the optimal pH values were changed to 10 for MB and 2 for AR88, resulting in a hydrogel formation. This might be indicated by the protonation of hydroxyl and amino groups resulting from a chitosan nitrate reaction in the AR88 solution.

## 1. Background

Excess nitrate in aquatic ecosystems has seriously endangered human health [1]. High nitrate concentrations in drinking water may cause methemoglobinemia or baby blue syndrome for infants and cancer [2,3,4,5,6]. The primary sources of nitrate are agricultural runoff [7], animal manure [8], leakage from septic tank systems [9], and industrial waste [10]. Due to its poor affinity for soil adsorption and high water solubility, nitrate is classified as the most widespread groundwater fomite in the world, presenting a danger to the safety of drinking water. As a result of the potential health hazards, the nitrate levels in drinking water are rigorously controlled in all nations. In Japan and the USA, the limit is 10 mg/L, but in China, it is 20 mg/L [1,11]. Additionally, nitrate is the primary cause of eutrophication [12]. Consequently, there is an urgent need to develop techniques and materials for removing excessive nitrate from water. Removing nitrate ions from water is one of the world’s biggest challenges.

Several techniques for nitrate removal have been described, such as ion exchange [13,14], biological [15,16], adsorption [17,18,19,20,21], electrodialysis [22], freezing–melting [23], reverse osmosis [24], and nanofiltration membrane [25] processes. They suggested that each of the techniques has advantages and limitations. For instance, the biological method is affordable; however, the residue of dead bacteria is problematic after processes, especially for use in drinking water [26,27]. Reverse osmosis and electrodialysis are expensive and produce nitrate waste brine [27]. The ion exchange method increases the corrosivity and aggressiveness of water by replacing SO_4_^2−^, HCO_3_, and Cl^−^ ions [28]. The advantage of this method is that the process could be used in either a small or big system. Among them, adsorption has been identified as one of the most desirable techniques for nitrate removal from aqueous solutions owing to its convenience, simple design, affordability, and ease of handling.

An essential part of the adsorption method is using a material product called an adsorbent. Nowadays, researchers have focused on biodegradable and non-toxic materials such as chitosan. Chitosan is obtained from chitin’s deacetylation, which has abundant functional groups (hydroxyl, amino, and methyl) and can easily react to or chemical modifications. However, chitosan has some limitations, including lower stability owing to its hydrophilicity and pH sensitivity [29]. Based on these reasons, cross-linked chitosan with zeolite is one of the best options to increase the stability of chitosan. Zeolites are crystalline aluminosilicates with porous structures and strong stability [30]. Some studies have successfully used zeolite/chitosan for the removal of dyes [31], heavy metals [32], humic acid [33], and nitrate [34]. However, the success of adsorption was based on the zeolite type and synthesis technique.

On the other hand, the ferric ion is also classified as chemically stable and can easily react with other materials such as biochar and chitosan for the removal of Cr(VI) [35], microcystin-LR [36], sulfamethoxazole [37], nitrate [38], and As(III) [39]. Based on the literature, we synthesized zeolite/chitosan/Fe^3+^ composites as a novel adsorbent to remove nitrate from water. Due to chitosan being soluble under acidic conditions, we used an alkaline treatment to obtain a solid adsorbent material through the use of NaOH. The effects of pH, adsorbent dosage, initial nitrate concentration, and contact time were investigated. Scanning electron microscopy (SEM), Fourier-transform infrared spectroscopy (FTIR), and pH of zero point of charge (pH_zpc_) were used for the characterization. In addition, we also evaluated the effects of competitive anions (SO_4_^2−^ and PO_4_^3−^) and dyes (methylene blue and acid red 88). The adsorption isotherm and kinetics were analyzed to describe the nitrate sorption mechanism.

## 2. Materials and Methods

### 2.1. Materials

Zeolite synthetic (ZL) was purchased from Tosoh Co. Ltd., 4560 Kaisei-Cho, Shunan City, Yamaguchi Prefecture, 746-8501, Japan. Chitosan (CH) was purchased from Acros Organics, Belgium. Ferric chloride (FeCl_3_.6H_2_O), Sodium hydroxide (NaOH), hydrochloric acid (HCl), acetic acid (CH_3_COOH), acid red 88 (AR88), Methylene blue (MB), and nitrate were purchased from Kanto Chemical Co. Inc. (Tokyo, Japan).

### 2.2. Adsorbent Preparation

The preparation of the adsorbent followed prior research with modifications [40]. Twenty-four hours were spent mixing 1 g of chitosan with 1% acetic acid (CH_3_COOH) in 100 mL. Two hours of mixing 0.5 g of zeolite with 25 milliliters of chitosan in an acetic acid solution. Then, 5 mL (0.1 M FeCl_3_) was added with rotary shaking for one hour (Rotator RT-50). After that, 25 mL of 1 M NaOH was added for 30 min. Then, the mixture was filtered and dried at 60 °C for 48 h. The adsorbent was then sieved at <100 µm. The adsorbent was ZLCH-Fe.

### 2.3. Adsorption Studies

All experiments were conducted three times with a mixing rotor (VMRC-5, As One), with an agitation speed of 100 rpm at 30 °C. We investigated the pH effect (2.5–10), adsorbent dosage (10–20 mg/50 mL), initial NO_3_ concentration (10–100 mg/L), and contact time (5–1380 min). The adsorption capacity and percent removal were calculated using Equations (1) and (2), respectively.

(1) $$q_{e/(t)}=\frac{C_{o}-C_{e}}{W}V$$

(2) $$\%\mathrm{Removal}=\frac{C_{o}-C_{e}}{C_{o}}100$$

where q_e:_ adsorption capacity (mg/g) (t: time); %Removal: removal efficiency (%); C_o_: initial NO_3_ concentration (mg/L); C_e_: NO_3_ equilibrium at the time (mg/L); W: adsorbent (ZLCH-Fe) mass (g); and V: Volume (L).

### 2.4. Characterization

Nitrate ions were determined using a nitrate test kit by spectrophotometry (Kyoritsu Chemical-Check Lab., Corp, Japan). The pH_zpc_ process followed previous research [40]. Before and after adsorption, the functional group of the adsorbent was analyzed by ATR-FTIR in an area of 400–4000 cm^−1^ (Thermo Scientific Nicolet iS10, Thermo Fisher Scientific Inc., Waltham, MA, USA). The photograph was examined using scanning electron microscopy (SEM) (Hitachi TM3000, Tokyo, Japan).

## 3. Results and Discussion

### 3.1. Morphology of ZLCH-Fe

**Figure 1:**
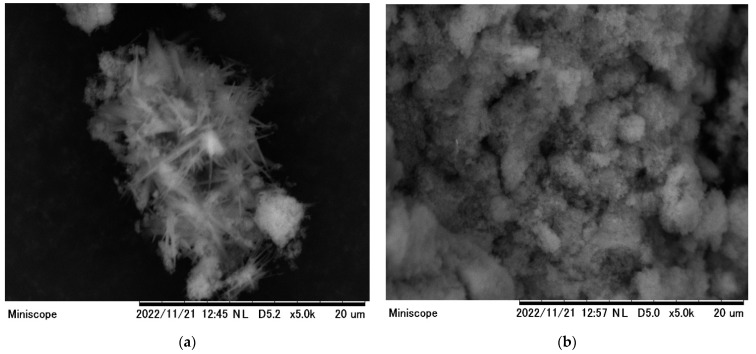
SEM images of ZLCH-Fe; (**a**) before nitrate adsorption; (**b**) after nitrate adsorption.

Figure 1 the SEM we used to evaluate the morphology of ZLCH-Fe before and after nitrate adsorption. Figure 1a demonstrates the interlayer, adhesive, porous morphologies, and needle-like structure on the surface. However, after adsorption, the surface texture was rough and needle-like disappeared (Figure 1b). This implies that the nitrate molecule has been absorbed by the ZLCH-Fe adsorbent.

### 3.2. FTIR Analysis

**Figure 2:**
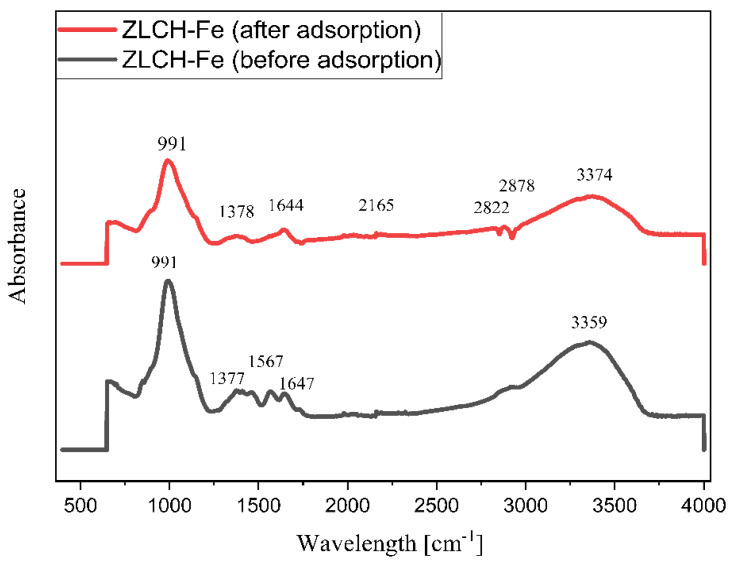
ATR-FTIR data before and after nitrate adsorption onto ZLCH-Fe.

Figure 2 shows the FTIR spectra of ZLCH-Fe before and after nitrate adsorption. Before the adsorption, the peak was at 3359 cm^−1^ and decreased to 3374 cm^−1^ after the adsorption process. This is because chitosan contains amine (-NH_2_) and hydrogen (-OH) groups in ZLCH-Fe and interacts with nitrate [41]. The other peak decreased after adsorption from 1647 to 1644 cm^−1^, corresponding to amide carbonyl and hydrogen bonding [42]. A peak at 1567 cm^−1^ corresponds to C=C stretching [43]. The increased band peak occurred after nitrate adsorption from 1377 to 1378 cm^−1^ was assigned to –CH bending to OH > -CHOH [44]. After nitrate adsorption, additional peaks formed at 2165, 2822, and 2878 cm^−1^, which are indicated by N-H stretching and S-CN (strong) and correlate to the band at 991 cm^−1^ (Si-O-Al or Al-O-Fe or Si-O-Fe).

### 3.3. The pH Effects and pH of Zero-Point Charge (pH_zpc_)

**Figure 3:**
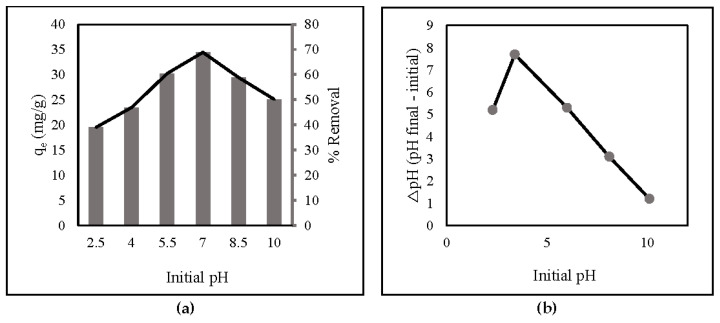
(**a**) Initial pH (10 mg of ZLCH-Fe; initial NO_3_: 10 mg/L; contact time: 30 min); (**b**) pH_zpc_.

The pH values significantly affect the adsorption capacity and percent of adsorbent removal because they are strongly connected to the ionization-induced electronic charges of functional groups on ZLCH-Fe [45]. From 2.5 to 10, the impact of initial pH on nitrate adsorption was investigated (Figure 3a). The surface charges of ZLCH-Fe were also assessed to explain the results (Figure 3b). The adsorption capacity and percent removal of nitrate increased as pH increased when the initial pH was less than 7 and decreased as pH increased when the initial pH was more than 7. This indicates that hydrogen, carboxyl, and amino groups are protonated at neutral pH (7) in ZLCH-Fe. These findings are similar to those reported by [1,45] for nitrate removal by FeMgMn-LDH and modified corn stalks, respectively.

### 3.4. The Effect of Adsorbent Dosage

**Figure 4:**
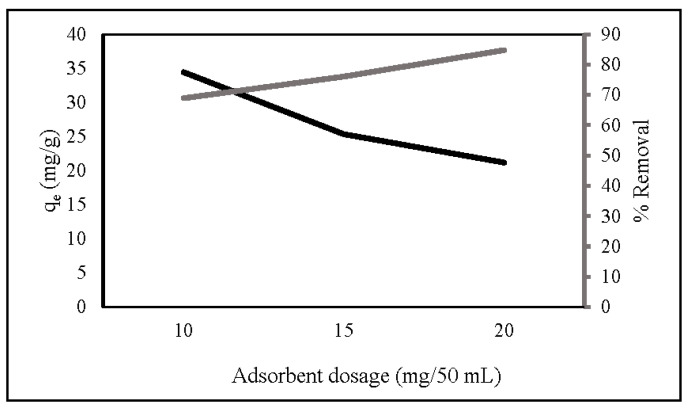
Effect of ZLCH-Fe dosage on the nitrate adsorption. (Initial NO_3_: 10 mg/L; contact time: 30 min; pH: 7.)

Adsorbent dosage provides active adsorption sites for nitrate removal. Figure 4 shows that the percentage of nitrate removal increased as the dosage increased from 10 mg/50 mL to 20 mg/50 mL, which may be attributed to the increase in accessible adsorption sites [39,46]. At a high adsorbent dosage, the value of adsorption capacity had the opposite result. This is due to overlapping adsorption sites decreasing the surface area [34,47]. At a low adsorbent dosage, all active sites were completely exposed, and the surface adsorption was rapidly saturated, indicating a high adsorption capability [48]. Based on the adsorption capacity, 10 mg ZLCH-Fe was selected as the optimal dosage.

### 3.5. The Effect of Initial Concentration

**Figure 5:**
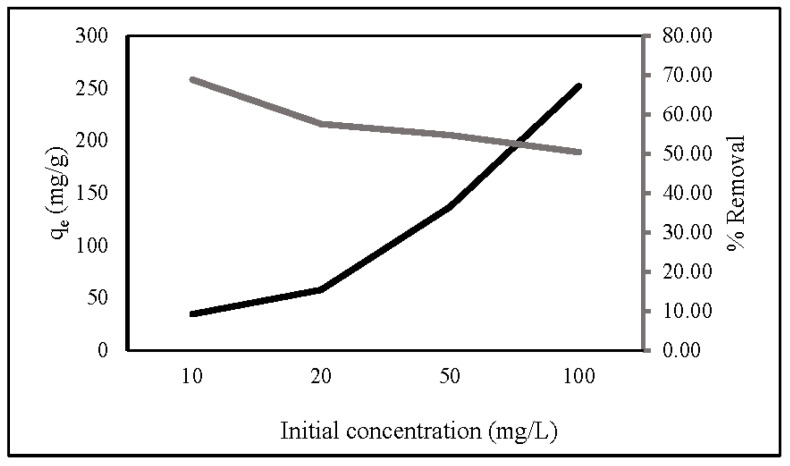
Effect of initial nitrate concentration on nitrate adsorption. (10 mg of ZLCH-Fe; contact time: 30 min; pH: 7).

The initial concentration is a key factor in determining the adsorption capacity and removal percentage of ZLCH-Fe. Figure 5 represents the adsorption capacity and percent removal of nitrate in the range of 10 to 100 mg/L initial nitrate concentration. We can see that the initial concentration increased; the adsorption capacity increased from 26.98 to 193.81 mg/g, contrary to the percent removal from 53.97 to 38.76%. This is because an increase in concentration would lead to an increase in the number of molecules, hence increasing the adsorption capacity. However, it would decrease the adsorbate’s mass transfer resistance. Consequently, the percent removal is decreased [39,49,50,51].

### 3.6. Isotherm Studies

At equilibrium in an adsorption mechanism, the adsorption isotherm adequately describes the distribution of adsorbate molecules between the solid and the liquid phases [52]. The adsorption data were analyzed using the Langmuir and Freundlich adsorption isotherms. Important information may be collected, such as the adsorption mechanism, the favorability of the adsorption process, and the adsorbate–adsorbent affinity. The Langmuir isotherm assumes a surface with uniform binding sites, comparable sorption energies, and no interaction between adsorbed species [39,53]. While the Freundlich isotherm revealed heterogeneous binding sites, this concept may be used to explain multilayer adsorption [54].

Linear correlation of the Langmuir in Equation (3).

(3) $$C_{e}{/q}_{e}=(\frac{C_{e}}{q_{\max}}{)+1/(K}_{1}q_{\max})$$

q_e_: the amount of the adsorbent (mg/g); K_l_: equilibrium constant of adsorption (L/mg); q_max_: maximal adsorption capacity; and C_e_: equilibrium concentration (mg/L).

The essential characteristics of the Langmuir isotherm may be represented in terms of the equilibrium parameter R_L_, a dimensionless constant also known as the separation factor in the following Equation (4).

(4) $$R_{L}=(\frac{1}{{1+\mathrm{bC}}_{o}})$$

C_o_: initial concentration (mg/L); R_L_: separation factor, indicating the adsorption is either R_L_ > 1 (unfavorable), R_L_ = 1(linear) or 0 < R_L_ (favorable).

Linear correlation of the Freundlich model in Equation (5).

(5) $${\ln\;q}_{e}{=\mathrm{lnK}}_{f}+\frac{1}{n}{\times \;\ln\;C}_{e}$$

K_f_: adsorption capacity (mg/g); 1/n: intensity of adsorption. C_e_ and q_e_ are the same as in the Langmuir model.

**Figure 6:**
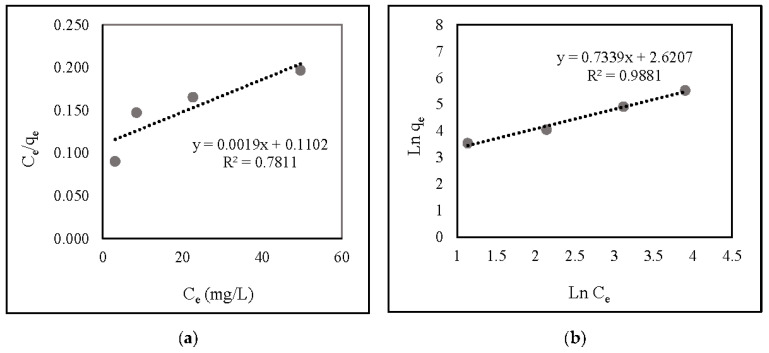
Linear isotherm plot of nitrate adsorption onto ZLCH-Fe; (**a**) Langmuir model; (**b**) Freundlich model.

Figure 6 and Table 1 illustrate the correlation and linear isotherm plots, respectively. The findings indicate that nitrate adsorption onto ZLCH-Fe was suitable with the Freundlich model (R^2^ = 0.9881) and favorable (R_L_ = 0.0011).

### 3.7. Kinetic Studies

**Figure 7:**
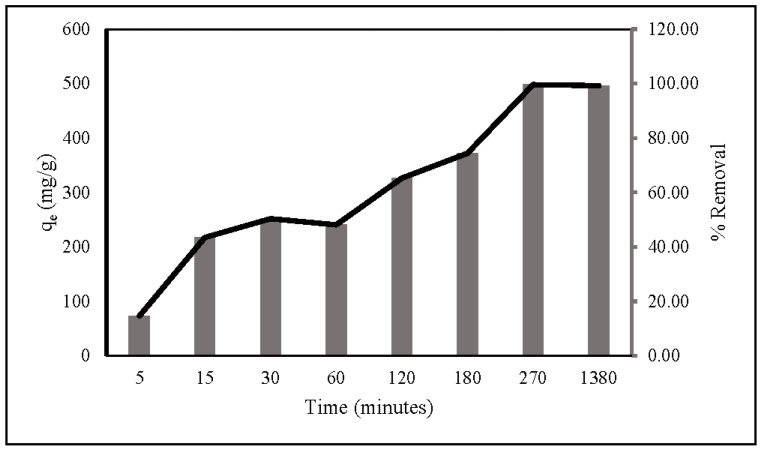
Contact times during nitrate adsorption. (10 mg of ZLCH-Fe; initial NO_3_: 100 mg/L; pH: 7.)

The kinetics of nitrate adsorption onto ZLCH-Fe were evaluated. Contact time is usually a factor in adsorption transformation processes. The interaction was investigated between 5 and 1380 min with 100 mg/L of nitrate concentration and 10 mg/50 mL at pH 7. As seen in Figure 7, adsorption capacity and percent removal rapidly increase in the first five minutes. Then, the gradual increase reached equilibrium at 270 min, at 498 mg/g and 99.64%, respectively.

It is essential to investigate the kinetics of adsorption because it explains the rate at which an adsorbent absorbs an adsorbate [55]. Pseudo-first-order and pseudo-second-order were used to explore the kinetics of adsorption [56,57]. Equations (6) and (7) provide the calculations for the first-order and second-order kinetic models, respectively.

(6) $${\log(q}_{e}-q_{t}{)=\mathrm{logq}}_{e}-K_{1}t$$

(7) $${t/q}_{t}{=1/(K}_{2}q_{e}^{2}{)+t/q}_{e}$$

where k_1_: rate constant of pseudo-first-order kinetic model (min^−1^); t: contact time (minutes). A linear plot of log t against log (q_e_ − q_t_) and t against t/q_t_ to determine K_1_ and K_2_ from the slope of linear plots, respectively.

**Figure 8:**
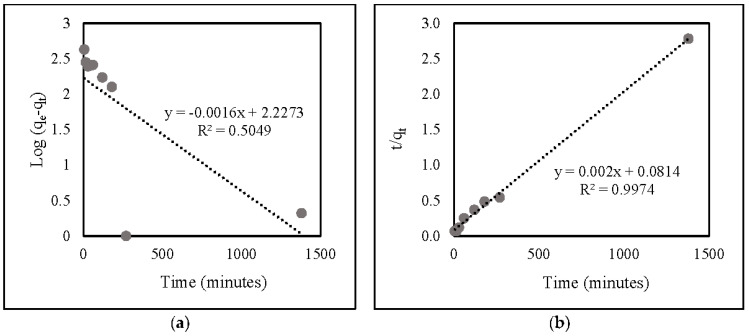
Linear kinetic plot of nitrate adsorption onto ZLCH-Fe: (**a**) pseudo-first-order model; (**b**) pseudo-second-order model.

Based on the correlation data (Table 2) and linear kinetic plot data (Figure 8), the R^2^ value of the second-order model (0.9974) was identical to that of the first-order one (0.5049). This suggests that nitrate adsorption onto ZLCH-Fe corresponds well to the pseudo-second-order model.

### 3.8. Anion Effect Studies

**Figure 9:**
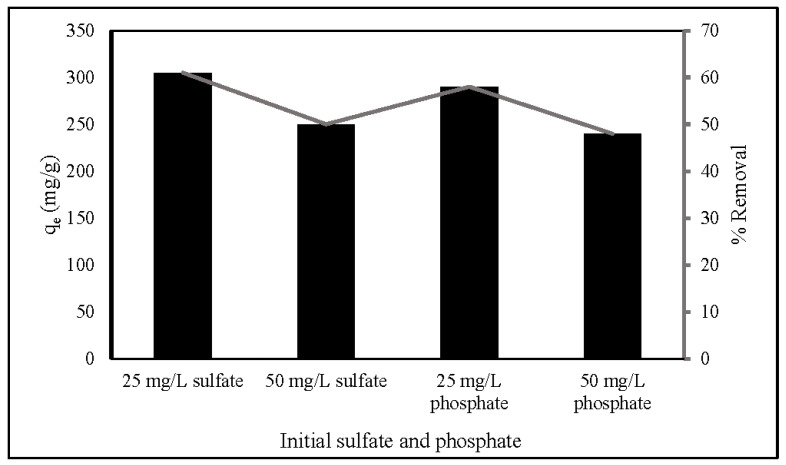
Effect of anions on nitrate adsorption. (10 mg of ZLCH-Fe; initial NO_3_: 100 mg/L; contact time: 270 min; pH: 7.)

Since there are several competing anions in an aqueous solution, it is essential to explore the influence of the anions. This study investigated the effect of SO_4_^2−^ and PO_4_^3−^ on nitrate adsorption by ZLCH-Fe, as shown in Figure 9. The process was carried out under optimal nitrate adsorption conditions (20 mg/50 mL (100 mg/L of NO_3_), pH 7 for 270 min at 30 °C). The adsorption capacity and percent removal in the absence of SO_4_^2−^ and PO_4_^3−^ were 498 mg/g and 99.64%, respectively. We can see that these anions affected adsorption capacity and percent removal of nitrate at the range of 240–305 mg/g and 48–61%, respectively. Increased initial SO_4_^2−^ and PO_4_^3−^ concentrations may inhibit nitrate adsorption. This is similar to the results of [1,20,58,59] used FeMgMn-LDH, calcined Mg-Al-Fe, natural zeolite, and Mg/Fe hydrotalcite, respectively.

### 3.9. Dye Effect Studies

**Figure 10:**
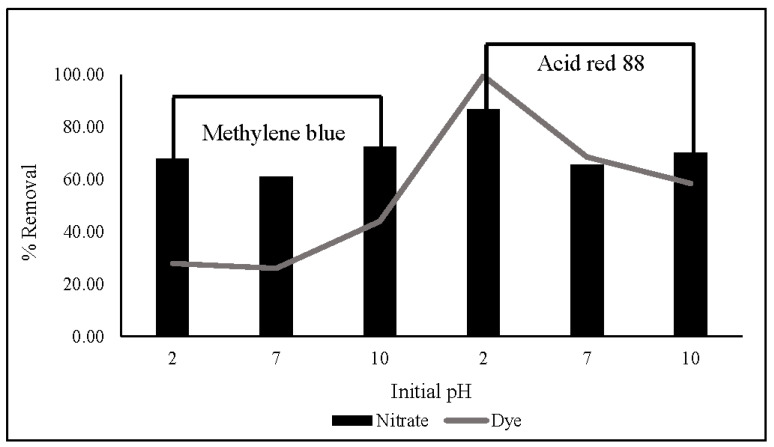
Effect of dyes (methylene blue and acid red 88) on nitrate and dye removal in different initial pH. (10 mg of ZLCH-Fe; initial NO_3_: 100 mg/L; initial dye: 25 mg/L; contact time: 5 min).

**Figure 11:**
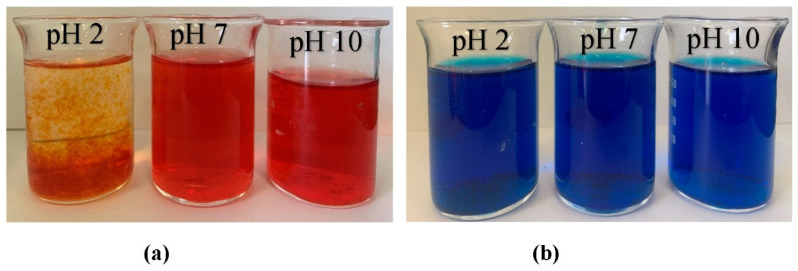
Photographs of dye solution after nitrate adsorption process (10 mg of ZLCH-Fe; initial NO_3_: 100 mg/L; initial dye: 25 mg/L; contact time: 5 min). (**a**) AR88; (**b**) MB.

The influence of dyes (MB and AR88) on nitrate removal is illustrated in Figure 10. The experiment was carried out using 10 mg of ZLCH-Fe, with an initial NO_3_ of 100 mg/L, and 25 mg/L of dyes at pH intervals of 2, 7, and 10 for 5 min in 50 mL. We can see that the highest dye percent removals at pH 10 for MB and pH 2 for AR88 were 44% and 99%, respectively. This result is similar to [40] and [49] for MB and AR88 removal, respectively. Interestingly, fact that when the solution was mixed with azo dye (AR88) at pH 2 (Figure 11a), the solution turned into hydrogels, followed by the maximum nitrate removal of 86.65%. This is due to chitosan solubility in acidic conditions [60]. AR88 is deprotonated, and more interactions are generated between Fe^3+^ and dyes. These results agree with [61] using nanoporous silica hydrogel by cross-linking SiO_2_-H_3_BO_3_-hexadecyltrimethoxysilane for azo dye removal. However, no hydrogels appeared to react with cationic dye (MB) in all pH ranges, as shown in Figure 11b.

**Figure 12:**
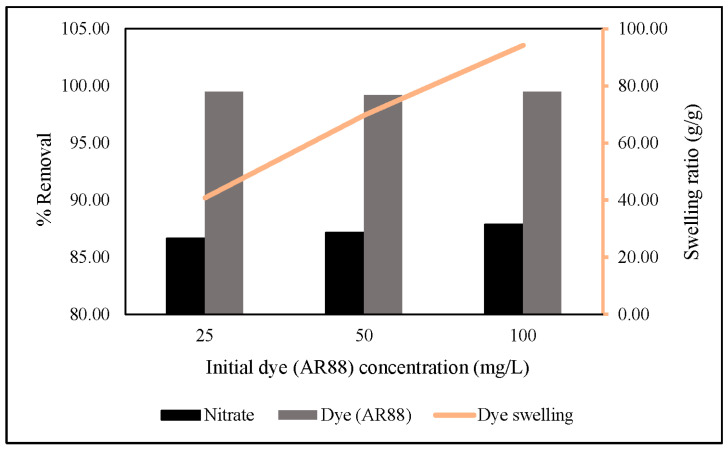
Effect of different initial dye (AR88) concentrations on nitrate and dye removal (10 mg of ZLCH-Fe; initial NO_3_: 100 mg/L; initial dye (AR88): 25, 50 and 100 mg/L; contact time: 5 min).

Thus, we increased the initial AR88 concentration up to 100 mg/L under the same conditions reported above. The results showed that an increased initial AR88 concentration would increase nitrate removal, followed by an increase in the swelling ratio, and there was no significant change in AR88 removal (Figure 12). The swelling ratio is followed in Equation (8) [49]. This indicates that azo dye group molecules are essential in capturing nitrate through hydrogel via ZLCH-Fe adsorption.

(8) $$\mathrm{Swelling}\;\mathrm{ratio}\;(g/g)=\frac{W_{w}-W_{d}}{W_{d}}$$

where W_w_: weight of swollen (before dry oven) (g); W_d_: weight of swollen (after dry oven) (g).

**Figure 13:**
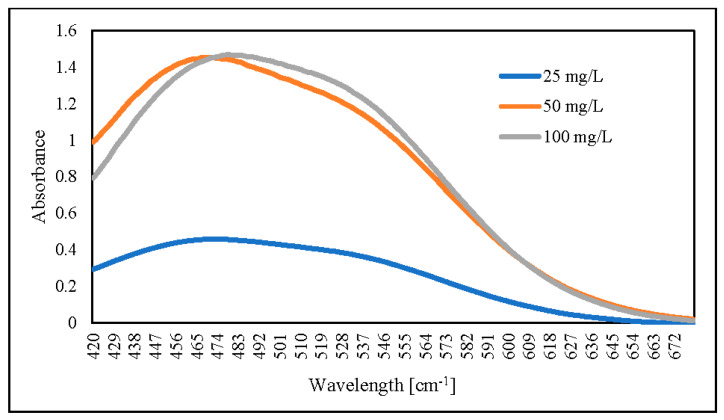
UV-visible spectra of AR88 dye hydrogel released by using 0.1 M NaOH (5 mg/20 mL, initial dye (AR88): 25, 50 and 100 mg/L; initial NO_3_: 100 mg/L; contact time: 60 min).

For the following steps, we dried hydrogel for 4 days at 60 °C. The dried hydrogel was mixed for 60 min at 30 °C with 0.1 M NaOH. The results showed that an increased initial AR88 concentration would increase absorbance (Figure 13). As compared, the absorbance peak decreased from 503 to 478 nm for AR88 original (AR88-Ori) and dried hydrogel (DH-ZLCH-Fe), respectively. Then, initial AR88 dye was 100 mg/L for further experiment.

**Figure 14:**
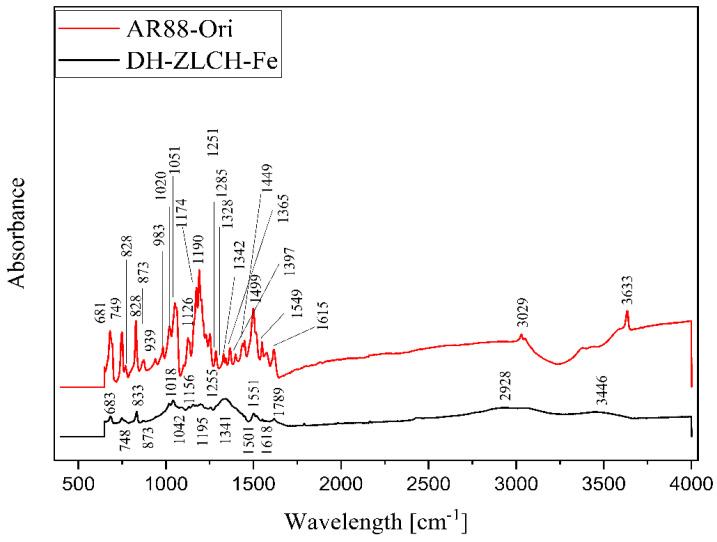
ATR-FTIR spectra of the AR88-Ori and DH-ZLCH-Fe.

Figure 14 shows the FTIR spectra of DH-ZLCH-Fe and AR88-Ori. Overall, the peak‘s area is almost similar. However, the peak spectra were changed. For example, the decreased peak of AR88-Ori to DH-ZLCH-Fe from 3633 to 3446 cm^−1^ and 3029 to 2928 cm^−1^, ascribed to –OH and C-H stretching, respectively [62]. The peak at 1780 cm^−1^ appeared in DH-ZLCH-Fe, corresponding to C=O stretching vibrations in COO- or COOCH_3_ [63]. This indicates that the carboxyl group of ZLCH-Fe reacted with azo dye (AR88). The appearance of the peak between 1650 and 1580 cm^−1^ is attributed to N-H bending. 1400 to 1600 cm^−1^ attributed to aromatic ring C=C stretching. The presence of peaks between 1251 and 1342 cm^−1^ may be attributed to S=O stretching. From 939 to 1195 cm^−1^ is corresponded to C-O-C stretching with or –CH-OH groups [64,65,66,67]. –CH_2_- and Si-O-Fe or Si-O-Al or Al-O-Fe groups may be associated with a peak between 681 and 983 cm^−1^ [63].

### 3.10. Comparison Nitrate Adsorption Capacity with Another Adsorbent

Table 3 shows the nitrate adsorption capacity of different adsorbents. We can see that ZLCH-Fe showed better adsorption than others. This indicates that ZLCH-Fe is a potential adsorbent to remove nitrate from water at either low or high concentrations.

## 4. Conclusions

This study examined the adsorption process of ZLCH-Fe as a promising adsorbent for nitrate removal from water. Experimental parameters such as pH, adsorbent dosage, initial nitrate concentration, and contact time were essential and investigated to determine the mechanism of nitrate adsorption. The results demonstrated that pH 7 is optimal. Increasing adsorbent dosage decreases adsorption capacity, while increasing initial nitrate concentration increases adsorption capacity. At 270 min, the adsorption capacity and percent removal were 498 mg/g and 99.64%, respectively. The isotherm and kinetic adsorption were compatible with the Freundlich and second-order models, respectively. The effects of anions (SO_4_^2−^ and PO_4_^3−^) and dyes (MB and AR88) were investigated. The results showed that the effect of anions might inhibit nitrate removal, in contrast with dye effects. When ZLCH-Fe interacted with AR88 at an initial pH of 2, hydrogels formed in the solution. In addition, the absorbance peak was decreased from 503 nm to 478 nm compared to the original AR88. Therefore, ZLCH-Fe might be utilized satisfactorily to remove nitrate from water.

## Figures and Tables

**Table 1:** Langmuir and Freundlich’s isotherm models of nitrate adsorption onto ZLCH-Fe.

q_e_ (exp)	Langmuir Parameters				Freundlich Parameters		
	q_max_	K_1_	R^2^	R_L_	K_f_	1/n	R^2^
498	526.42	9.0726	0.7810	0.0011	417.52	0.7338	0.9881

**Table 2:** Pseudo-first-order and Pseudo-second order kinetics models of nitrate adsorption onto ZLCH-Fe.

Pseudo-First Order			Pseudo-Second Order		
q_e_	K_1_	R^2^	q_e_	K_2_	R^2^
9.274	−5.9259 × 10^−6^	0.5049	500	4.914 × 10^−5^	0.9974

**Table 3:** Comparison of the nitrate adsorption capacity of several adsorbents.

Adsorbent	Adsorption Capacity (mg/g)	References
FeMgMn-LDH	10.56	[1]
Mg–Al hydrotalcite	34.36	[4]
Chitosan/Zeolite Y/Nano ZrO_2_	23.58	[10]
Mg-Al-Fe hydrotalcite	123.3	[20]
Chitosan/zeolite molecular sieves	2.11	[34]
Chitosan-Fe^3+^ complex	8.35	[38]
Modified corn stalks	24.20	[45]
Fe_3_O_4_/ZrO_2_/chitosan nanocomposite	89.3	[68]
Layered double hydroxide	117.8	[69]
Modified carbon residue	80	[70]
Dolochar	6.51	[71]
ZLCH-Fe	498	Present study

## Data Availability

Not applicable.
